# Digital pathology-aided assessment of tumor-infiltrating T lymphocytes in advanced stage, HPV-negative head and neck tumors

**DOI:** 10.1007/s00262-020-02481-3

**Published:** 2020-01-24

**Authors:** Emma J. de Ruiter, Reinout H. de Roest, Ruud H. Brakenhoff, C. René Leemans, Remco de Bree, Chris H. J. Terhaard, Stefan M. Willems

**Affiliations:** 1 Department of Pathology, University Medical Center Utrecht, Heidelberglaan 100, 3584 CX Utrecht, The Netherlands; 2 Department of Otolaryngology/Head and Neck Surgery, Cancer Center Amsterdam, Amsterdam University Medical Center, Amsterdam, The Netherlands; 3 Department of Head and Neck Surgical Oncology, University Medical Center Utrecht, Utrecht, The Netherlands; 4 Department of Radiotherapy, University Medical Center Utrecht, Utrecht, The Netherlands

**Keywords:** Head and neck squamous cell carcinoma (HNSCC), Tumor-infiltrating lymphocytes (TILs), T cells, Prognostic biomarkers

## Abstract

**Aim:**

This study aimed to evaluate the presence and prognostic value of tumor-infiltrating T cells in the tumor epithelium in advanced stage, HPV-negative head and neck squamous cell carcinoma (HNSCC) patients treated with primary chemoradiotherapy using digital pathology.

**Methods:**

Pre-treatment biopsies from 80 oropharyngeal, 52 hypopharyngeal, and 29 laryngeal cancer patients were collected in a tissue microarray (TMA) and immunohistochemically stained for T-cell markers CD3, CD4, CD8, FoxP3, and PD1, and for immune checkpoint PD-L1. For each marker, the number of positive tumor-infiltrating lymphocytes (TILs) per mm^2^ tumor epithelium was digitally quantified and correlated to overall survival (OS), disease-free survival (DFS), and locoregional control (LRC), as well as to clinicopathological characteristics. Differences in clinical outcome were estimated using Cox proportional hazard analysis and visualized using Kaplan–Meier curves.

**Results:**

The patient cohort had a 3-year OS of 58%, with a median follow-up of 53 months. None of the T-cell markers showed a correlation with OS, DFS or LRC. A low N stage was correlated to a better prognosis (OS: HR 0.39, *p* = 0.0028, DFS: HR 0.34, *p* =  < 0.001, LRC: HR 0.24, *p* = 0.008). High TIL counts were more often observed in PD-L1-positive tumors (*p* < 0.05).

**Conclusion:**

This study showed an objective, digital pathology-aided method to assess TILs in the tumor epithelium. However, it did not provide evidence for a prognostic role of the presence of CD3 + , CD4 + , CD8 + , FoxP3 + , and PD1 + TILs in the tumor epithelium of advanced stage, HPV-negative HNSCC patients treated with primary chemoradiotherapy.

**Electronic supplementary material:**

The online version of this article (10.1007/s00262-020-02481-3) contains supplementary material, which is available to authorized users.

## Introduction

Despite the improvement of treatment outcome with the use of radiotherapy in combination with concomitant chemotherapy, an estimated 25–50% of all head and neck squamous cell carcinoma (HNSCC) patients still face locoregional recurrence and overall survival remains poor [1]. Failure of locoregional control of HNSCC strongly contributes to morbidity and mortality [2, 3]. Identifying robust biomarkers predicting patients at risk for recurrent disease after therapy would be of great value in selecting the best treatment for each individual patient [4].

For many types of cancer, it has become clear that the interplay between tumor cells and their microenvironment strongly influences tumor aggressiveness and therapy resistance [5, 6]. Therapies targeting the anti-tumor immune response are rapidly evolving and are already implemented in a variety of cancer types [7]. In HNSCC, immune checkpoint inhibitors nivolumab and pembrolizumab are recently incorporated in clinical practice, with other immunotherapeutic agents probably soon to follow [8].

Many studies indicated the presence of tumor-infiltrating lymphocytes (TILs) in the tumor microenvironment to be a prognostic factor for treatment outcome in different types of cancer [9]. Especially T cells have been studied extensively in this context [10–12]. In HNSCC, several studies showed a prognostic favorable role for several subtypes of tumor-infiltrating T cells [13]. Also in patient cohorts exclusively treated with primary radiotherapy with or without concomitant chemotherapy, the presence of T cells was correlated to a better treatment outcome. Especially high infiltration with CD3 + and CD8 + TILs appeared to be a prognostic favorable characteristic; the role of CD4 + and FoxP3 + TILs was less clear [14–17]. The CD8/FoxP3 ratio has also been suggested as a promising, potential biomarker [15, 18, 19]. However, as far as we know, this ratio has not been examined in this specific patient group before.

A limitation of many prognostic biomarker studies in HNSCC is the use of heterogeneous patient cohorts with respect to treatment modality, tumor stage, tumor subsite and HPV status and/or a small number of study subjects. Furthermore, consensus on robust cutoffs is lacking, because the method of assessing TILs varies strongly among studies [13].

In this study, we aimed to assess the presence and prognostic value of CD3 + , CD4 + , CD8 + , FoxP3 + , and PD1 + TILs, and the CD8/FoxP3 ratio in the tumor epithelium and its relation to prognosis. To do so, we used a relatively large patient cohort consisting of advanced stage, HPV-negative head and neck squamous cell carcinoma patients treated with chemoradiotherapy and an objective, digital pathology-aided scoring system.

## Materials and methods

### Patients and clinical data

This study was conducted using a consecutive, retrospective cohort of patients with HNSCC treated at the University Medical Center Utrecht (UMCU), Utrecht, and the Amsterdam UMC (location VUmc), between January 2009 and December 2014. Inclusion criteria were (1) stage III or IV, HPV-negative oropharyngeal, hypopharyngeal, and laryngeal squamous cell carcinoma, (2) treatment with radiotherapy with concomitant cisplatin or carboplatin with curative intent, and (3) availability of tumor tissue and clinical data on survival outcomes. Patients treated with surgical resection of the tumor, or having distant metastases, a medical history of radiotherapy in the head and neck area, or a prognosis-affecting double tumor or prior malignancy were excluded.

For each patient, the following clinical data were collected: age, sex, performance state, comorbidity, prior malignancies, tobacco and alcohol usage, tumor localization, tumor stage, T stage, N stage, total radiation dose, and total chemotherapy dose. Comorbidity was scored using the Adult Comorbidity Evaluation-27 (ACE-27) [20]. Performance state was scored using the WHO classification [21].

### Treatment protocol

Standard treatment regimen existed of a total radiation dose of 70 Gy on the primary tumor and positive lymph nodes in 35 fraction of 2 Gy, and a total dose of 46–57.75 Gy on the elective lymph nodes, in combination with cisplatin in a total dose of 300 mg/m^2^ body surface area in three divided doses every 3 weeks.

### Tissue microarray construction and immunohistochemistry

From all included patients, formalin-fixed, paraffin-embedded (FFPE) pre-treatment biopsies were collected. Sections of the FFPE blocks were stained with hematoxylin and eosin (H&E) and assessed by a dedicated head and neck pathologist (S.M. Willems) to mark representative tumor regions. For each patient, three 0.6 mm tissue cores were obtained from the assigned area of the FFPE blocks and collected in a tissue microarray (TMA). The TMA was constructed by a fully automated tissue microarray instrument, as described before [22].

TMA tissue sections (4 µm) were immunohistochemically stained with antibodies for the following antigens: CD3 (A452; 1:200; DAKO), CD4 (SP35, 1:25; Cellmarque), CD8 (CD8/144B; 1:100; DAKO), FoxP3 (236A/E7; 1:750; Abcam), PD1 (NAT105; 1:100; Abcam), and PD-L1 (SP263, Ventana RTU). Staining was performed using a Ventana Bench Mark XT Autostainer (Ventana Medical Systems, Tucson, AZ, USA).

### HPV detection

All cases included in this study were HPV-negative. Tumors were considered HPV-negative if less than 70% of tumor cells stained positive for p16 INK4a by immunohistochemistry (JC8, 1:1200, Immunologic). P16-positive tumors were tested for the presence of HPV-DNA by PCR and were excluded if high-risk HPV-DNA was detected [22, 23].

### Digital immunohistochemical analysis

**Fig. 1:**
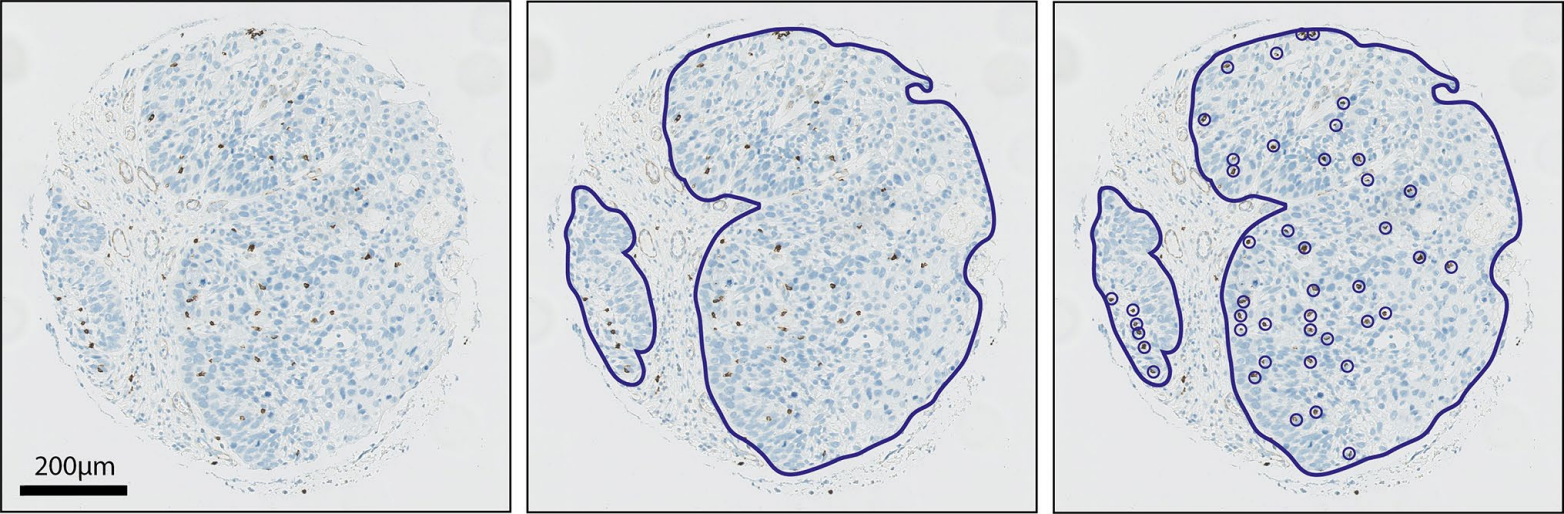
Method of digital quantification of TILs. **a** Representative image of a TMA core. **b** For each TMA core, the tumor epithelium was annotated. **c** Positively stained TILs were quantified within the annotated area

Stained sections of the TMA were digitalized using Aperio Scanscope XT slide scanner at a magnification of 40× resulting in a resolution of 0.233 microns per pixel. For each TMA core, the tumor epithelium was annotated and quantified using Imagescope 12.1 (Fig. 1). Within the annotated area, positively stained lymphocytes were counted. PD-L1 was scored positive if the mean percentage of stained tumor cells from the three TMA cores was more than 5%; cells with any membranous staining were considered positive [24]. The immunohistochemical analysis was performed by a head and neck researcher (E. J. de Ruiter) and a dedicated head and neck pathologist (S. M. Willems), who were blinded for clinical outcome. Discrepancies were resolved by consensus. The two observers scored 50 TMA cores separately to calculate interobserver variability.

### Statistical analysis

For each T-cell marker, the number of positive TILs per mm^2^ tumor epithelium was calculated by dividing the summed number of lymphocytes of the three corresponding TMA cores by the total tumor epithelium area of the three cores. Tumors were excluded from analysis if less than two TMA cores were assessable or if the total annotated tumor area was less than 0.1 mm^2^.

Intraclass correlation coefficients (ICC) between different TMA cores from the same patient were calculated using SPSS (SPSS statistics 23) based on a mean-rating (*k* = 3), absolute-agreement, two-way mixed-effects model (Koo 2016) [25].

The number of positive TILs/mm^2^ was correlated to overall survival (OS), disease-free survival (DFS) and locoregional control (LRC). OS was defined as the number of days between the first day of treatment and the date of death, DFS as the number of days between the first day of treatment and the date of recurrence of disease or the date of death, and LRC as the number of days between the first day of treatment and the date of local or regional recurrence. Patients without an event were censored at the date of their last visit to the clinic.

Correlations between TIL counts and clinical variables were assessed by Mann–Whitney *U* tests for dichotomous clinical variables, Kruskal–Wallis tests for clinical variables stratified in more than two groups, and Spearman correlation for continuous clinical variables. Correlations with OS, DFS, and LRC were assessed using Cox proportional hazards regression in R (× 64 3.3.2) using the survival and survminer packages. To perform the regression analysis, TIL counts were log transformed by taking their log_2_. The predictive value of each T-cell marker was visualized by Kaplan–Meier curves comparing tumors with high and low TIL counts stratified by the median value; HRs and *p*-values accompanying the Kaplan–Meier curves were calculated using logrank tests.

## Results

### Patient characteristics

A total of 161 patients were eligible for inclusion, among which 80 were oropharyngeal, 52 hypopharyngeal, and 29 laryngeal cancer patients. The patient cohort had a 3-year OS of 58%, with a median duration of follow-up of 53 months. Clinical characteristics of the patient cohort are summarized in Table 1.

**Table 1:** Patient characteristics

Hospital	VUmc	97	(60.2%)
	UMC Utrecht	64	(39.8%)
Age	Mean (SD)	59.2	(6.7)
Sex	Male	106	(65.8%)
	Female	55	(34.2%)
WHO	0	38	(23.6%)
	1	94	(58.4%)
	2	7	(4.3%)
	Unknown	22	(13.7%)
ACE-27	None (0)	58	(36.0%)
	Mild (1)	78	(48.4%)
	Moderate (2)	24	(14.9%)
	Severe (3)	1	(0.6%)
Prior malignancy		13	(8.1%)
	HNSCC	3	(1.9%)
	Other	10	(6.2%)
Tobacco usage	Current	120	(74.5%)
	Former	35	(21.7%)
	Never	5	(3.1%)
	Unknown	1	(0.6%)
Packyears	Mean (SD)	40.3	(19.3)
Alcohol usage	Current	118	(73.3%)
	1–3/day	51	(31.7%)
	≥ 4/day	67	(41.6%)
	Former	28	(17.4%)
	Never	14	(8.7%)
	Unknown	1	(0.6%)
Tumor location	Oropharynx	80	(59.7%)
	Hypopharynx	52	(32.3%)
	Larynx	29	(18.0%)
T stage	T1	3	(1.9%)
	T2	28	(17.4%)
	T3	62	(38.5%)
	T4a	52	(32.3%)
	T4b	16	(9.9%)
N stage	N0	24	(14.9%)
	N1	21	(13.0%)
	N2a	11	(6.8%)
	N2b	46	(28.6%)
	N2c	53	(32.9%)
	N3	5	(3.1%)
	Unknown	1	(0.6%)
Stage	III	28	(17.4%)
	IVa	121	(75.2%)
	IVb	20	(12.4%)
Chemotherapy completed	Yes	119	(73.9%)
	Switch	23	(14.3%)
	No	19	(11.8%)
Treatment outcome	No recurrence	101	(62.7%)
	Residu/recurrence	60	(37.3%)
	Locoregional	40	(24.8%)
	Distant	30	(18.6%)

Almost all patients were treated with radiotherapy in combination with cisplatin. Five patients were treated with carboplatin instead of cisplatin. 24 patients were initially treated with cisplatin, but switched to carboplatin due to adverse events. 19 patients discontinued treatment after two doses of cisplatin, receiving a total dose of 200 mg/m^2^ body surface area.

### Immunostaining of TILs on pre-treatment biopsies

**Fig. 2:**
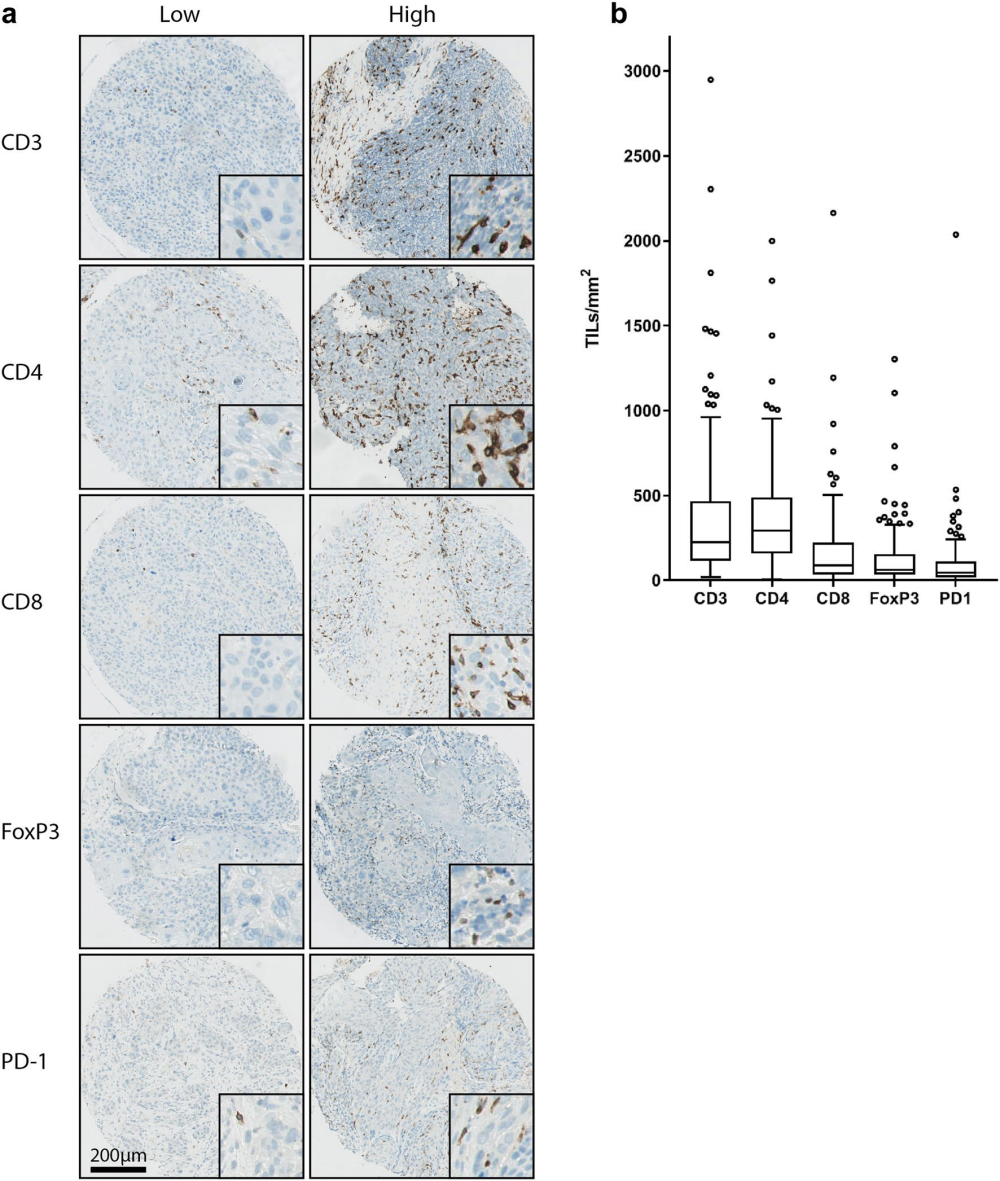
Variability of number of TIL subsets. **a** Representative images of TMA cores containing low and high numbers of CD3 + , CD4 + , CD8 + , FoxP3 + and PD1 + TILs. **b** Boxplot diagrams of the number of TILs/mm^2^ tumor epithelium

Figure 2a shows representative images of TMA cores containing low and high numbers of TILs. The distribution of the data is visualized in boxplot diagrams for each T-cell marker (Fig. 2b). Details on median values and ranges of TIL counts in TILs/mm^2^ for each biomarker and median values and ranges of the log_2_-transformed TIL counts are shown in Supplementary Table 1.

Some tissue cores were lost during processing or did not contain any tumor epithelium. If less than two out of three TMA cores of one tumor were assessable or if the total annotated tumor area was less than 0.1 mm^2^, tumors were excluded from analysis of that specific marker.

Concordance between TMA cores from the same patients was good for CD3 (intraclass correlation coefficient (ICC): 0.86, 95% confidence interval (CI) 0.80–0.90), CD8 (ICC: 0.84, 95% CI 0.78–0.89), FoxP3 (ICC: 0.80, 95% CI 0.72–0.86), and PD1 (ICC: 0.87, 95% CI 0.81–0.91) and moderate to good for CD4 (ICC: 0.69, 95% CI 0.57–0.79). Interobserver variability was generally very low (Supplementary Table 2).

### Correlation between TILs and clinicopathological characteristics

PD-L1 positivity of the tumor was correlated to a high amount of CD3 + (*p* = 0.047), CD4 + (*p* = 0.021), CD8 + (*p* = 0.038) and PD1 + (*p* = 0.014) TILs; Furthermore, a correlation was found between PD1 + TILs and comorbidity. Patients with an ACE-27 score of none to mild were more likely to have a high PD1 + TIL count than patients with a score of moderate to severe (*p* = 0.012). All correlations between TILs and clinicopathological characteristics are shown in Supplementary Table 3.

### Correlation between TILs and treatment outcome

**Fig. 3:**
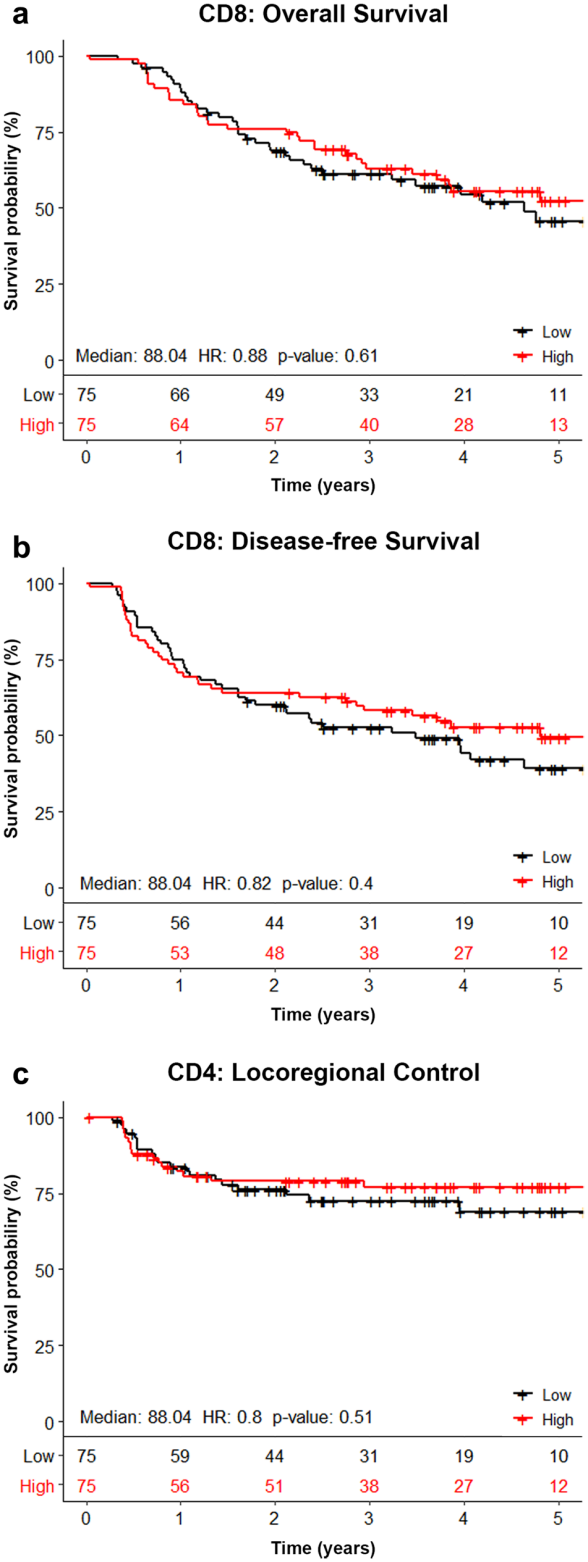
Association between the number of CD8 + TILs and clinical outcome. Kaplan–Meier curves visualizing the association between the number of CD8 + TILs in the tumor epithelium and OS (**a**), DFS (**b**), and LRC (**c**). The median number of CD8 + TILs was used as cutoff for the survival analysis. No association was found between the number of CD8 + TILs and OS, DFS, or LRC

The outcome of all survival analyses is shown in Table 2. No significant correlations were found between any of the TIL markers and OS, DFS, or LRC. Correlations between CD8 + TILs and treatment outcome were visualized in Kaplan–Meier curves (Fig. 3). Kaplan–Meier curves of the other biomarkers are shown in Supplementary Figs. 1–5.

**Table 2:** Univariate analysis of the correlation between biomarkers and OS, DFS and LRC

Marker	Comparison	No of cases	HR	95% CI	*p* value
Overall survival					
CD3	Per 1 increase (log_2_)	152	0.95	(0.82–1.11)	0.53
CD4	Per 1 increase (log_2_)	149	0.96	(0.80–1.16)	0.68
CD8	Per 1 increase (log_2_)	150	0.95	(0.84–1.07)	0.40
FoxP3	Per 1 increase (log_2_)	154	0.96	(0.83–1.12)	0.59
PD1	Per 1 increase (log_2_)	141	0.92	(0.81–1.04)	0.17
CD8FoxP3ratio	Per 1 increase (log_2_)	146	0.98	(0.84–1.13)	0.76
PD-L1	< 5% vs ≥ 5%	158	1.27	(0.75–2.14)	0.38
Tumor location	Larynx	161	Ref.		
	Oropharynx		1.94	(0.94–4.00)	0.074
	Hypopharynx		1.40	(0.64–3.04)	0.40
T stage	T1–3 vs T4	161	0.79	(0.50–1.26)	0.33
N stage	N0–1 vs N2–3	161	0.39	(0.21–0.72)	**0.0028**
Age	Per year increase	161	1.00	(0.96–1.03)	0.89
Sex	Male vs Female	161	1.21	(0.73–2.00)	0.46
ACE-27	< 2 vs ≥ 2	161	0.75	(0.40–1.39)	0.35
WHO	< 2 vs ≥ 2	149	0.65	(0.36–1.18)	0.15
Disease-free survival					
CD3	Per 1 increase (log_2_)	152	0.95	(0.82–1.10)	0.49
CD4	Per 1 increase (log_2_)	149	0.97	(0.81–1.15)	0.72
CD8	Per 1 increase (log_2_)	150	0.94	(0.84–1.04)	0.23
FoxP3	Per 1 increase (log_2_)	154	0.99	(0.86–1.13)	0.84
PD1	Per 1 increase (log_2_)	141	0.91	(0.81–1.03)	0.12
CD8FoxP3ratio	Per 1 increase (log_2_)	146	0.92	(0.80–1.06)	0.24
PD-L1	< 5% vs ≥ 5%	158	1.34	(0.82–2.20)	0.25
Tumor location	Larynx	161	ref		
	Oropharynx		1.50	(0.79–2.85)	0.21
	Hypopharynx		1.21	(0.61–2.39)	0.59
T stage	T1–3 vs T4	161	0.93	(0.60–1.44)	0.73
N stage	N0–1 vs N2–3	161	0.34	(0.19–0.62)	** < 0.001**
Age	Per year increase	161	1.00	(0.97–1.03)	0.84
Sex	Male vs Female	161	1.18	(0.74–1.88)	0.49
ACE-27	< 2 vs ≥ 2	161	0.89	(0.49–1.61)	0.70
WHO	< 2 vs ≥ 2	149	0.69	(0.40–1.20)	0.17
Locoregional control					
CD3	Per 1 increase (log_2_)	152	1.03	(0.83–1.28)	0.77
CD4	Per 1 increase (log_2_)	149	1.02	(0.77–1.33)	0.92
CD8	Per 1 increase (log_2_)	150	1.01	(0.85–1.19)	0.92
FoxP3	Per 1 increase (log_2_)	154	1.14	(0.92–1.40)	0.23
PD1	Per 1 increase (log_2_)	141	0.96	(0.80–1.14)	0.62
CD8FoxP3ratio	Per 1 increase (log_2_)	146	0.92	(0.75–1.13)	0.43
PD-L1	< 5% vs ≥ 5%	158	1.71	(0.78–3.72)	0.18
Tumor location	Larynx	161	ref		
	Oropharynx		2.23	(0.77–6.47)	0.14
	Hypopharynx		1.70	(0.55–5.26)	0.36
T stage	T1–3 vs T4	161	0.85	(0.45–1.60)	0.61
N stage	N0–1 vs N2–3	161	0.24	(0.086–0.69)	**0.008**
Age	Per year increase	161	1.01	(0.96–1.06)	0.61
Sex	Male vs female	161	1.17	(0.59–2.32)	0.66
ACE-27	< 2 vs ≥ 2	161	1.17	(0.46–3.00)	0.75
WHO	< 2 vs ≥ 2	149	0.25	(0.075–0.81)	**0.021**

The correlation between biomarkers and OS, DFS, and LRC was assessed in a Cox proportional hazards regression. TIL counts and CD8/FoxP3 ratio were log transformed prior to the regression. The prognostic value of biomarkers is expressed as hazard ratios (HR), 95% confidence intervals (95% CI) and *p* values. None of the T-cell markers showed a significant association with OS, DFS, or LRC. Patients with an N stage ≤ 1 showed a better OS, DFS and LRC than patients with an N stage of ≥ 2. A lower WHO performance state was correlated to a better LRC

Statistically significant *p*-values (values below 0.05) are denoted in bold

Due to lack of correlation between TIL counts and survival data, no multivariate analysis was performed.

### Correlation between clinicopathological characteristics and treatment outcome

The only clinical variable correlated to OS and DFS was N stage. N0 and N1 patients showed a significantly better OS (HR 0.39, 95% CI 0.21–0.72, *p* = 0.0028) and DFS (HR 0.34, 95% CI 0.19–0.62, *p* =  < 0.001) than N2 and N3 patients. N stage and WHO performance state were correlated to LRC. Patients with a low N stage had an increased LRC (HR 0.24, 95% CI 0.086–0.69, *p* = 0.008), as did patients with a WHO performance score below 2 (HR 0.25, 95% CI 0.075–0.81, *p* = 0.021).

## Discussion

In this study, we assessed the presence and prognostic value of CD3, CD4, CD8, FoxP3, and PD1 positive TILs, as well as the CD8/FoxP3 ratio, in the head and neck tumor epithelium in pre-treatment biopsies of HNSCC patients using an objective, digital pathology-aided method.

In the last decades, it has become clear that the immune system plays an indispensable role in tumor development and progression [26]. It has therefore been a major target for the development of new treatment strategies, resulting in the implementation of various immunotherapeutic options in different types of cancer [27–29]. Also in HNSCC, the results of immunotherapy in recurrent and metastatic disease are promising [8, 30].

Previous studies have provided evidence that the presence of an immune response prior to treatment could enhance the effect of radiotherapy and chemotherapy [31–33], suggesting that the presence of TILs could be used as a predictive biomarker for treatment outcome. Indeed, in many types of cancer, the presence of immune cells in the tumor microenvironment was associated with a better treatment outcome [9].

The anti-tumor immune response is a complex process, involving various players of the innate and adaptive immune system [34, 35]. In this study, we examined the role of the T cell, the most studied subtype as it is able to directly target tumor cells. However, different subsets of T cells with different functions exist. We assessed TILs expressing CD3, CD4, CD8, FoxP3 and PD1.

First, a correlation was observed between infiltration of TILs and PD-L1 expression in the tumor: PD-L1-positive tumors showed higher CD3 + , CD4 + , CD8 + and PD1 + TIL counts in the tumor epithelium than PD-L1-negative tumors, a phenomenon that was observed before in HNSCC and in other types of cancer [36–38]. It is an important observation that PD-L1 expression is more often observed in highly infiltrated head and neck tumors, because it suggests that these tumors might be likely to benefit from immunotherapy targeting the PD1/PD-L1 interaction [39].

Second, we assessed the prognostic value of CD3 + , CD4 + , CD8 + , FoxP3 + , and PD1 + TILs in HNSCC. Several studies showed a prognostic favorable role for the presence of T cells. However, the literature on the prognostic role of TILs in HNSCC assessed by immunohistochemistry predominantly comprised small studies, using heterogeneous patient cohorts, providing insufficient data to draw robust conclusions on subgroups [13]. Methods differ strongly among studies and are not always clearly described, hindering consensus on cutoff values and implementation of TILs as predictive biomarkers in clinical practice. Furthermore, studies using TCGA datasets showed a prognostic favorable effect of immune cell profiles in HNSCC as well [40], but RNA-sequencing data do not tell in which compartment of the tumor the immune cells are located, while the prognostic effect of TILs in the tumor epithelium might differ from the effect of TILs in the tumor stroma [41].

In this study, we used a relatively large patient cohort, with a high homogeneity regarding treatment modality, tumor stage, and HPV status, in which we assessed the presence of T cells in the tumor epithelium using an objective method. Given all technical and clinical optimizations in our study design, we did not find a prognostic role for T-cell markers CD3, CD4, CD8, FoxP3, PD1 and the CD8/FoxP3 ratio in the head and neck tumor epithelium, an observation that is in contrast with previous studies [13].

A possible explanation for these findings may lie in the specificity of our patient cohort regarding treatment modality and HPV status. All patients in the study cohort were diagnosed with advanced stage HNSCC and were exclusively treated with chemoradiotherapy, while most studies assessing the prognostic value of T cells also included surgically treated patients [42–49]. It was shown that (chemo)radiotherapy affects the tumor microenvironment and is able to enhance the anti-tumor immune response in rectal and pancreatic cancer [50–54]. This could mean that the pre-treatment composition of the tumor microenvironment is of less importance than the anti-tumor immune response induced by the (chemo)radiotherapy. However, this is not supported by multiple studies that do show an association between the pre-treatment presence of TILs and treatment outcome [14, 55, 56].

Also, we exclusively included patients with HPV-negative head and neck tumors. According to several studies, a more prominent immune response is observed in HPV-positive tumors compared to HPV-negative tumors, and some studies suggested that TILs play a more important role in HPV-positive tumors than in HPV-negative tumors [15, 48, 57, 58], which might explain the lack of prognostic value of T-cell markers in our patient cohort. However, there are also studies that suggest the opposite [14, 59].

Another important remark in the light of our results is the fact that we specifically assessed T cells in the tumor epithelium. Stromal T cells have been shown to have their effect on prognosis and treatment outcome as well and it was suggested that the prognostic significance of intra-epithelial and stromal TILs differs [41]. It might be possible that the prognostic value of T cells in the tumor microenvironment is completely explained by their presence in the tumor stroma. This was also suggested by Oguejiofor et al., who used a similar patient cohort [17]. However, in our study, a tissue microarray was used for staining and quantifying TILs and the amount of tumor stroma varied strongly among the different cores. Therefore, assessing TILs in the tumor stroma was not attempted.

Lastly, this study used TMAs, which only comprise a part of the tumor biopsy and might not adequately represent the original tumor. However, three cores were taken per patient, which should take account of heterogeneity within the tumor biopsy [60]. A bigger restraint might be the fact that the researchers were limited in the usage of patient material in the first place. As the primary treatment was chemoradiotherapy and not surgery, only small pre-treatment biopsies from the periphery of the tumor were available for research. Immune cell infiltration has been shown to differ between different parts of the tumor, which might explain the discrepancy we found with studies that assessed complete resected tumor lumps. However, it is inherent to the organ-sparing nature of primary chemoradiotherapy that only a small part of the tumor tissue is available for examination, which not only limits research, but has to be taken into consideration in diagnostics as well.

In conclusion, this study did not provide evidence for a prognostic value of the presence of CD3 + , CD4 + , CD8 + , FoxP3 + , and PD1 + T lymphocytes in the tumor epithelium of advanced stage, HPV-negative HNSCC patients treated with primary chemoradiotherapy. However, an objective method to assess TILs in the tumor epithelium was described.

### Electronic supplementary material

Below is the link to the electronic supplementary material.

Supplementary file1 (PDF 603 kb)

## Abbreviations

ACE-27: Adult Comorbidity Evaluation-27
AUC: Area under the curve
CI: Confidence interval
DFS: Disease-free survival
FFPE: Formalin fixed, paraffin embedded
HR: Hazard ratio
ICC: Intraclass correlation coefficient
LRC: Locoregional control
ROC: Receiver operating characteristics
TMA: Tissue microarray
UMC: University Medical Center
VUmc: Vrije Universiteit Medical Center
