# Supplementary material for: Digital pathology-aided assessment of tumor-infiltrating T lymphocytes in advanced stage, HPV-negative head and neck tumors
Source: Cancer Immunol Immunother. 2020 Jan 24;69(4):581–91. doi: 10.1007/s00262-020-02481-3 (PMC7113229; doi:10.1007/s00262-020-02481-3)
Supplement: Supplementary file 1 — Supplementary file1 (PDF 603 kb) [file 262_2020_2481_MOESM1_ESM.pdf]

SUPPLEMENTARY FIGURE 1

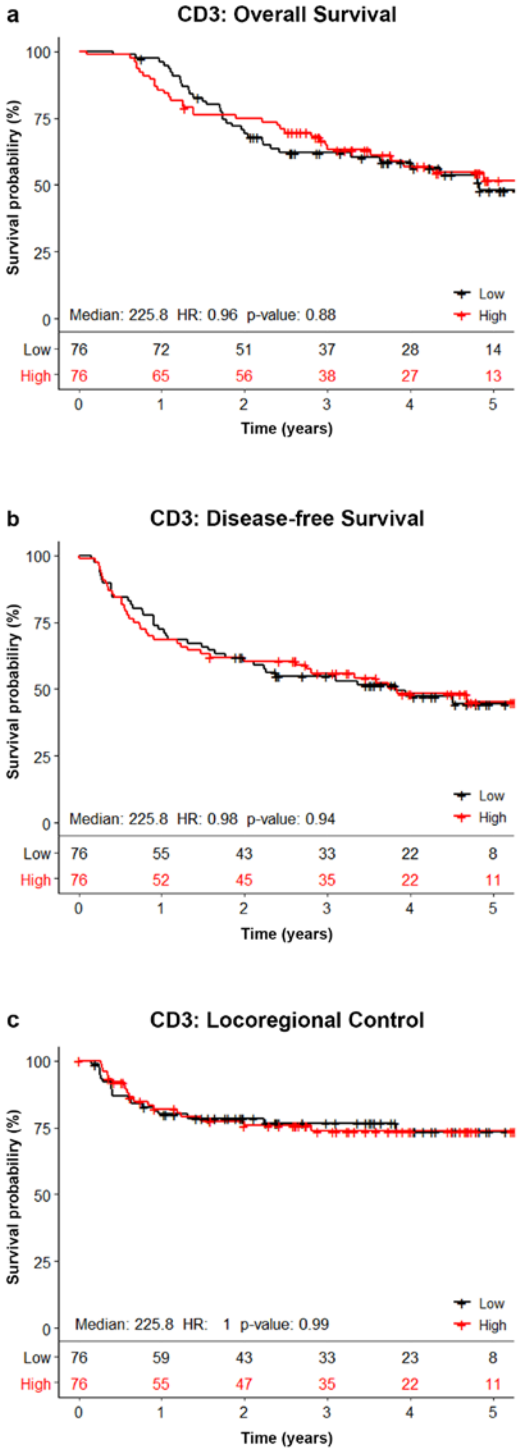

Supplementary figure 1: Association between the number of CD3+ TILs and clinical outcome. Kaplan-Meier curves visualizing the association between the number of CD3+ TILs in the tumor epithelium and OS (**a**), DFS (**b**), and LRC (**c**). The median number of CD3+ TILs was used as cut-off for the survival analysis. No association was found between the number of CD3+ TILs and OS, DFS, or LRC.

SUPPLEMENTARY FIGURE 2

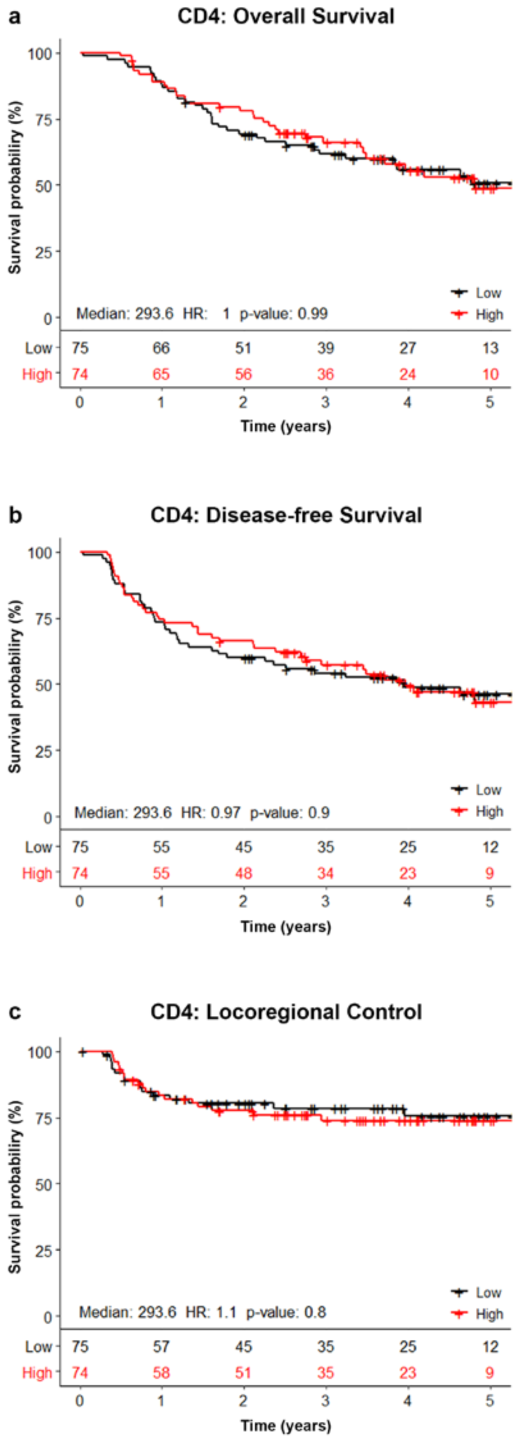

Supplementary figure 2: Association between the number of CD4+ TILs and clinical outcome. Kaplan-Meier curves visualizing the association between the number of CD4+ TILs in the tumor epithelium and OS (**a**), DFS (**b**), and LRC (**c**). The median number of CD4+ TILs was used as cut-off for the survival analysis. No association was found between the number of CD4+ TILs and OS, DFS, or LRC.

SUPPLEMENTARY FIGURE 3

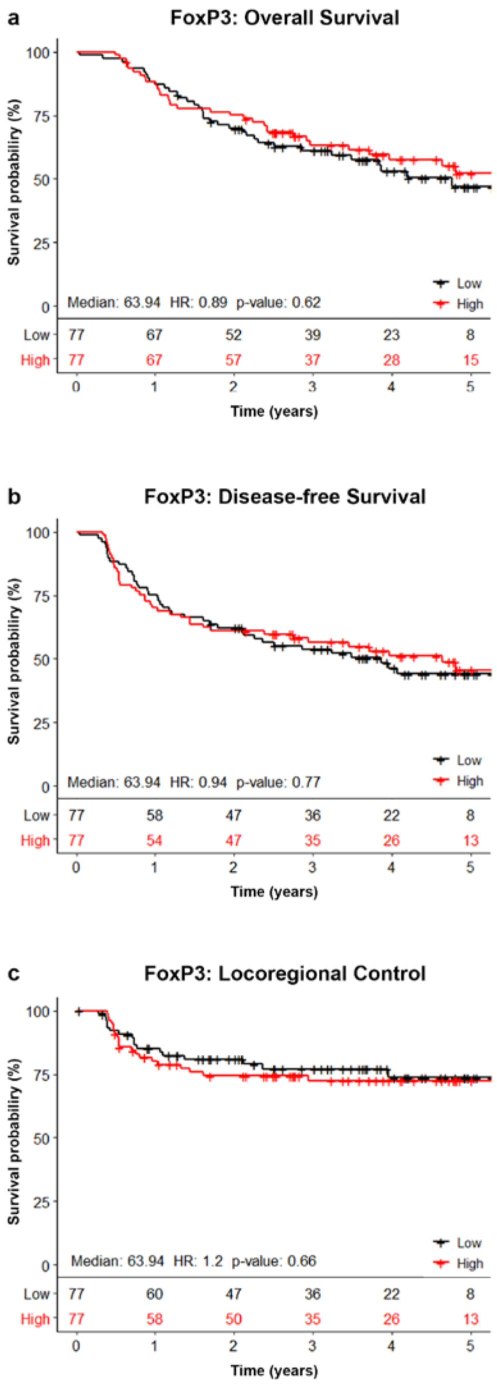

Supplementary figure 3: Association between the number of FoxP3+ TILs and clinical outcome. Kaplan-Meier curves visualizing the association between the number of FoxP3+ TILs in the tumor epithelium and OS (**a**), DFS (**b**), and LRC (**c**). The median number of FoxP3+ TILs was used as cut-off for the survival analysis. No association was found between the number of FoxP3+ TILs and OS, DFS, or LRC.

SUPPLEMENTARY FIGURE 4

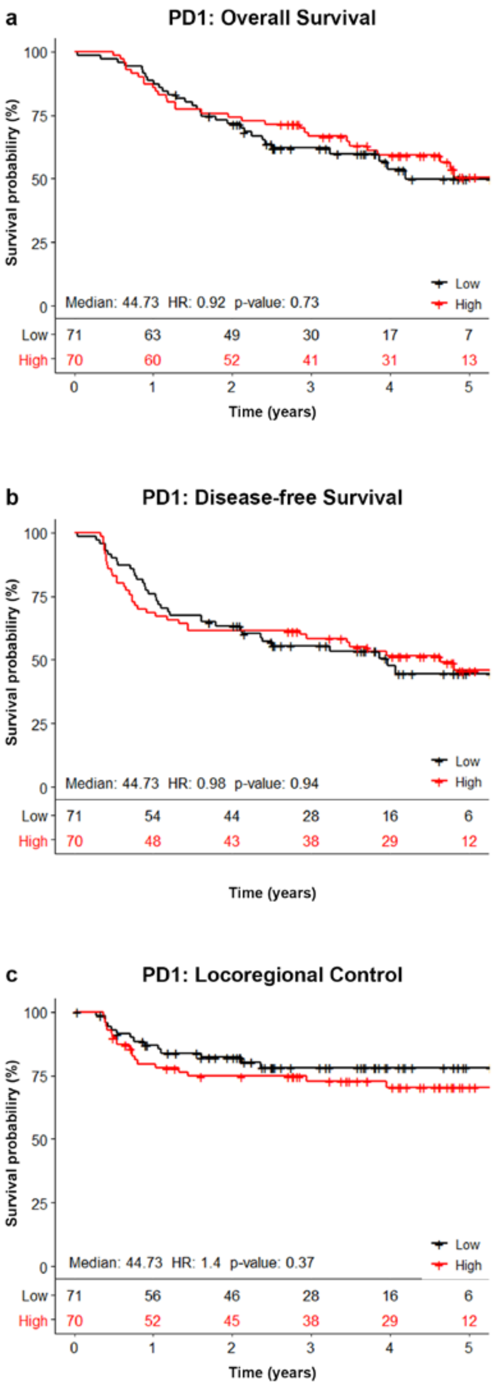

Supplementary figure 4: Association between the number of PD-1+ TILs and clinical outcome. Kaplan-Meier curves visualizing the association between the number of PD1+ TILs in the tumor epithelium and OS (**a**), DFS (**b**), and LRC (**c**). The median number of PD1+ TILs was used as cut-off for the survival analysis. No association was found between the number of PD1+ TILs and OS, DFS, or LRC.

SUPPLEMENTARY FIGURE 5

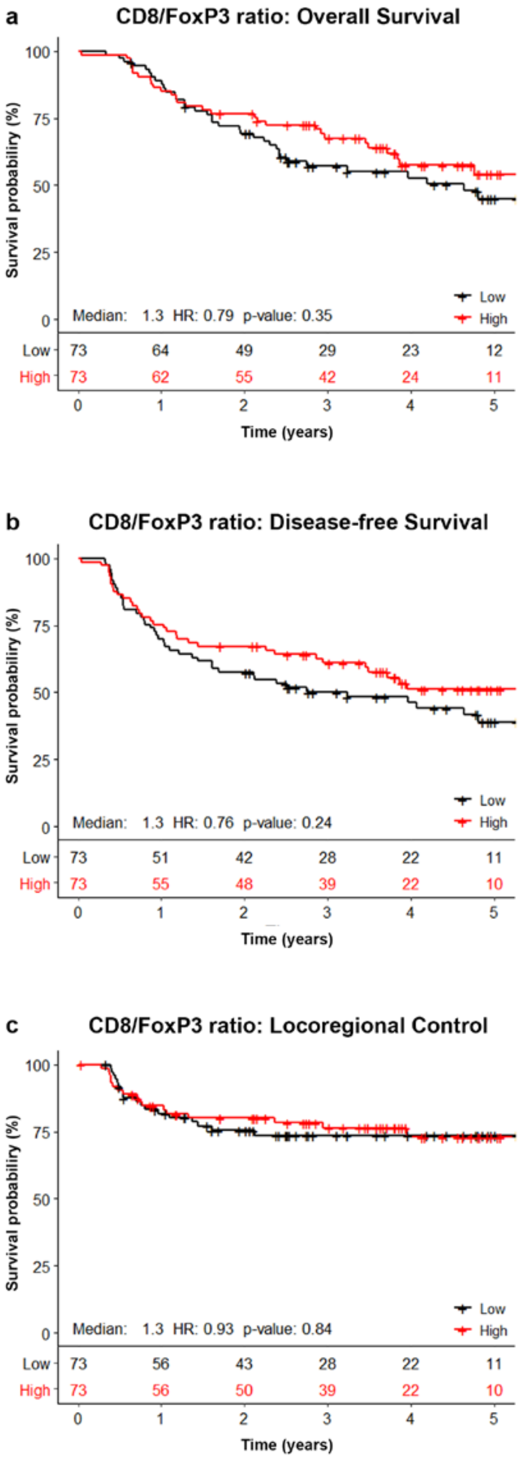

Supplementary figure 5: Association between the number of CD8/FoxP3 ratio and clinical outcome. Kaplan-Meier curves visualizing the association between the number of the CD8/FoxP3 ratio in the tumor epithelium and OS (**a**), DFS (**b**), and LRC (**c**). The median number of the CD8/FoxP3 ratio was used as cut-off for the survival analysis. No association was found between the number of the CD8/FoxP3 ratio and OS, DFS, or LRC.

SUPPLEMENTARY TABLE 1

	Median [TILs/mm^2]	Interquartile range	Median log_2(TILcount)	Interquartile range log_2(TILcount)
**CD3**	217	[115 – 455]	7.82	[6.86 – 8.84]
**CD4**	261	[153 – 476]	8.20	[7.35 – 8.92]
**CD8**	83	[33 – 230]	6.46	[5.15 – 7.81]
**FoxP3**	63	[36 – 129]	6.02	[5.20 – 7.21]
**PD-1**	41	[16 – 107]	5.52	[4.21 – 6.77]
**CD8/FoxP3 ratio**	1.32	[0.55 – 2.98]	0.38	[-0.89 – 1.32]

Supplementary Table 1: Median values and interquartile ranges of TIL counts (TILs/mm^2] and the log_2 of TIL counts..

SUPPLEMENTARY TABLE 2

Marker	ICC (95% CI)
**CD3**	0.990 (0.971-0.996)
**CD4**	0.986 (0.975-0.992)
**CD8**	0.998 (0.997-0.999)
**FoxP3**	0.903 (0.794-0.950)
**PD1**	0.949 (0.856-0.977

Supplementary Table 2: Inter observer analysis. Intraclass correlation coefficients were calculated based on a mean-rating (k = 2), absolute-agreement, 2-way mixed-effects model.

SUPPLEMENTARY TABLE 3

	CD3	p	CD4	p	CD8	p	FoxP3	p	PD1	p
**Age**										
	r = 0.00	0.99	r = 0.04	0.64	r = 0.00	0.98	r = 0.07	0.39	r = 0.02	0.80
**Sex**										
Male	229 [125-453]	0.78	311 [173-490]	0.62	100 [43-226]	0.11	59 [36-125]	0.47	49 [19-128]	0.093
Female	208 [110-484]		263 [157-448]		65 [27-196]		77 [36-176]		26 [15-103]	
**ACE-27**										
None/mild	231 [118-473]	0.23	309 [169-485]	0.35	98 [42-230]	0.14	68 [36-166]	0.62	50 [19-121]	**0.012**
Moderate/severe	209 [107-307]		248 [125-483]		60 [29-179]		61 [39-113]		19 [12-44]	
**WHO**										
<2	339 [112-579]	0.71	366 [222-509]	0.48	97 [39-221]	0.52	89 [38-166]	0.78	53 [15-102]	0.82
≥2	224 [129-490]		297 [171-491]		99 [43-282]		66 [39-173]		40 [18-122]	
**Tumor location**										
Oropharynx	200 [118-386]	0.72	293 [163-467]	0.87	65 [35-194]	0.30	62 [31-116]	0.36	37 [17-93]	0.48
Hypopharynx	292 [109-557]		316 [215-481]		109 [37-225]		75 [41-221]		57 [15-121]	
Larynx	256 [125-453]		264 [123-497]		125 [43-327]		62 [43-130]		51 [20-107]	
**T stage**										
T1-3	278 [132-496]	0.12	318 [178-495]	0.20	97 [33-229]	0.95	69 [39-142]	0.29	45 [17-107]	0.99
T4	194 [112-384]		280 [142-446]		80 [43-211]		54 [32-165]		45 [18-120]	
**N stage**										
N0-1	279 [112-588]	0.54	301 [157-468]	0.84	104 [52-298]	0.23	63 [34-149]	0.92	40 [12-129]	0.72
N2-3	210 [118-453]		294 [171-504]		80 [35-209]		64 [36-145]		45 [19-106]	
**PD-L1**										
<5%	216 [115-392]	**0.047**	280 [157-425]	**0.021**	76 [42-386]	**0.038**	62 [36-121]	0.19	38 [15-91]	**0.014**
≥5%	366 [121-797]		423 [173-705]		172 [42-386]		74 [36-237]		68 [26-132]	

Supplementary Table 3: Correlations between TILs and clinicopathological characteristics. The correlation between T cell markers and age was assessed using Spearman correlation (Spearman's r and p-value in table), the correlation between T cell markers and tumor location was assessed using Kruskal-Wallis test (median, interquartile ranges and p-value in table), all other correlations were calculated using Mann-Whitney U tests (median, interquartile ranges and p-values in table).
